# Energy Efficient Multicast Communication in Cognitive Radio Wireless Mesh Network

**DOI:** 10.3390/s22155601

**Published:** 2022-07-27

**Authors:** Imran Baig, Najam Ul Hasan, Prajoona Valsalan, Manaf Zghaibeh

**Affiliations:** ECE Department, Dhofar University, Salalah 211, Oman; ibaig@du.edu.om (I.B.); pvalsalan@du.edu.om (P.V.); mzghaibeh@du.edu.om (M.Z.)

**Keywords:** cognitive radio network, wireless mesh network, multicasting, bipartite graph

## Abstract

Multicasting is a basic networking primitive used in a wide variety of applications that is also true for cognitive radio-based networks. Although cognitive radio technology is considered to be the most promising technology to deal with spectrum scarcity, it relates to completely different aspects of networking and presents new challenges. For cognitive radio-based multicast sessions, it is important to use the spectrum efficiently by reducing the number of channels used as well as engaging fewer nodes in data relaying. This will benefit the network in three ways. First, it will decrease the number of transmissions. Second, it will help to reduce energy usage. Third, it will spare more channels and relay nodes for simultaneous multicast sessions. To achieve these advantages, efficient channel selection and relay nodes are required based on hop-to-hop communication. In this paper an algorithm has been developed that attempts to minimize energy consumption by selecting the minimum possible number of relay nodes and channels for a multicast session, taking into account the sporadic availability of the spectrum. The proposed method performs effectively compared to the flooding method in terms of energy consumption for the provided examples in multicasting.

## 1. Introduction

Multicasting is a fundamental networking primitive that is used by a wide range of applications. The fundamental idea of multicasting is to facilitate group communication by allowing the transmission and routing of packets to various destinations while using as few resources as possible, such as energy consumption, necessary bandwidth, and relay nodes. However, with the rise of low-power gadgets, efficient resource usage has become even more crucial. Cognitive radio technology has been recognized as a key enabler for next-generation wireless communication and effective spectrum usage. This helps us to minimize energy usage for transmission even further. This technology allows the nodes equipped with a cognitive radio to utilize the unused portion of the licensed spectrum in an opportunistic manner. The availability of these unused portions of the spectrum is dependent on the usage pattern of the licensed user. This varies in time and location, due to which a cognitive radio may observe a different set of available channels (which is referred to as spectrum heterogeneity). This raises several challenges for cognitive radio networks [1].

This paper is interested in the multicast problem in cognitive radio wireless mesh network (CR-WMNs). Although a lot of research has already been done on multicasts in wireless networks, these findings cannot be applied due to the spectrum heterogeneity associated with CR-WMNs. This is because the properties of this kind of network may result in the transmission of the same frame multiple times over multiple channels depending on the availability of the channels at different nodes. Furthermore, most of the previous work done, which will be discussed later, focused towards either improving the throughput or decreasing the latency of the transmission for multicast session which is not applicable for an environment with low power devices. Keeping in view the current proliferation of low power devices, we considered the reduction of energy consumption to be the prime objective of this work. In this work, how to minimize these multiple transmissions of the same frame in order to reduce the energy consumption for multicast communication in CR-WMNs has been studied. To have a better understanding of multimedia communication over the CR-WMNs, a survey article presented by Amjad et al. in [2] had been referred to.

For this purpose, first, an energy consumption model has been developed for multicast communication and an optimization problem designed to construct an energy efficient multicast tree while considering the spectrum heterogeneity property of CR-WMNs. Subsequently, rather than developing a single layer approach that solely focuses on multicast routing, a cross-layer heuristic approach is proposed to solve this optimization problem. The proposed approach considers the routing and channel assignment mechanism to construct an energy efficient multicast tree in CR-WMNs. In the proposed technique, for a given multicast session, a node level is defined for each node in the CR-WMN. The node level is determined on the basis of the node’s distance in terms of the number of hops from the source node (with the farthest node being assigned the highest level). A mechanism to select the forwarding nodes and assign channels to these nodes is then introduced for each level. This mechanism starts from the highest to the lowest level and ultimately results in the construction of a multicast tree. The decision to select the forwarding nodes and assign channels to these nodes is carried out in such a way that a frame is transmitted a minimum number of times to reach all its intended receiving nodes while taking care of spectrum heterogeneity. The main contribution of this paper can be summarized as follows:

1. A multicasting problem for CR-WMNs is established to reduce the amount of energy used during a multicast session.
2. A multicast routing algorithm is proposed to choose the multicast path from source nodes to destination nodes while minimizing the number of relaying nodes and channels required.
3. The simulation results for the proposed algorithm are presented in terms of the number of relaying nodes needed, the number of channels used, and the total amount of energy used.

The remaining paper is organized as follows. Section 2 discusses the previous work related to multicasting in CR-WMNs. Section 3 presents the network model and the minimum energy multicast tree construction problem for CR-WMNs. Section 4 presents the proposed cross-layer approach to the construction of an energy efficient multicast tree for CR-WMNs. Section 5 presents simulation results in order to demonstrate the performance of the proposed technique. Finally, Section 6 concludes the paper.

## 2. Related Work

Energy efficient multicasting in wireless networks is a multi-faceted problem that can be addressed in several ways, to name a few, including optimization of transmission power [3], optimization of routing paths [4], and spectrum and bandwidth optimization for multicast sessions [5]. CR is considered a promising candidate technology to be used in this field. However, these results in [3,4,5] are not applicable to the CRN because of its unique features in terms of spectrum availability. Thus, it is essential to develop new protocols and algorithms for multicasting in CRNs.

Over the last few years, many studies have been reported on multicast communication in CRNs. A multipath on-demand routing protocol for CRNs has been presented in [6]. The protocol is based on the Artificial Intelligence Reinforcement Learning Method and aims to increase throughput and reduce end-to-end delays. Results have shown that, particularly in the case of high primary user (PU) activity, the proposed protocol is superior to current state-of-the-art protocols.

A multicast scheduling technique for a single cell CR-WMN is proposed in [7]. In this technique, client nodes assist the base station in delivering the multicast data over orthogonal channels to reduce total multicast time. Network coding is also utilized for a further reduction in overall multicasting time. The authors of [8] have also used network coding for multicasting in CRNs with the goal of minimizing total transmission cost (amount of transmitted data) while taking into account both channel uncertainty and node mobility. A random network coding-based multicasting strategy is presented in [9], and the aim was again to reduce the total number of transmissions (amount of data).

An optimization method for multicast scheduling in CRNs using network coding was presented in [10]. In this optimization problem, several considerations, including protection of the PU and fairness, were taken into account. Two methods were proposed: a greedy method based on centralized optimization and a stochastic optimization-based online method. An optimization problem is presented in [11] which jointly defines multicast routes and channel assignments with the goal of increasing multicast throughput in multi-hop CRNs. In [12], the implementation of multicast non-orthogonal multiple access (NOMA) in CRNs is explored. Significant performance gains were achieved by CR user scheduling through cooperative NOMA. Other multicasting strategies inspired by NOMA are proposed for CRNs [13,14].

A multicast tree construction method is proposed in [15] such that the total bandwidth used by the multicast tree is reduced to meet the predetermined *Quality of Service* (QoS) requirements. The authors presented two approaches to solve this problem based on a minimal spanning tree so that time slots are allocated to tree links in a manner that reduces bandwidth usage. A minimum tree-based multicast routing protocol is proposed in [16]. The contribution of this paper was to increase the total throughput and the delivery rate of the packets.

The authors of [17] presented a problem related to joint power and rate allocation with a goal of maximizing the average multicast rate in CRNs while guaranteeing QoS in terms of the probability of service failure. A hidden node problem for multichannel multicast transmission is raised in [18], and a local multicast concept is introduced to address this issue. A channel assignment algorithm is presented for this purpose that considers both partially overlapping as well as orthogonal channels. The authors in [19] proposed a multi-channel multicast problem for a multi-radio network of wireless mesh nodes with directional antennas. The goal was to reduce interference by selecting the best multicast session tree, channel, and antenna beams. A survey article [20] has been referred to for a more detailed review of multicast communication in CRNs. In Table 1, the above related work is summarized as follows.

## 3. Network Model and Problem Formulation

Consider a CR-WMN with N nodes. First, each node employs a spectrum sensing technique to identify the channels unused by the PU. In this work, any specific spectrum sensing technique was not considered as it is beyond the scope of this work. Once each node *i* performs spectrum sensing, it comes up with a set of available channels *C_i_* unused by the PUs. The available channels for two nodes may be different from each other depending on their geographical location. Furthermore, it is also assumed that each node shares its spectrum sensing results with its neighboring nodes via a control channel. The control channel setup is also beyond the scope of this work. It is assumed that each node transmits with its maximum transmission power P and has a transmission range *R_T_*. Two nodes *i* and *j* are said to be neighbors if the distance d*_ij_* between them is less than *R_T_* and *C_i_* ∩ *C_j_* ≠ Ø. There is a set *K* of multicast sessions. Each multicast session *k* ∈ *K* consists of a source node and multiple destination nodes. For each session, a multicast routing tree is constructed rooted at the source node. The multicast tree consists of source, forwarding/relay, and destination nodes. The multicasting on a multicast tree can be broken down into a number of local forwarding operations. For each forwarding operation, a hyperlink *H*(*i*, *h*, *I*) is established over channel *h*, where *i* is the forwarding node and *I* is the set of receiving nodes. The energy consumed by any node *i* involved in a hyperlink for a duration of bit τ can be modelled as

(1) $$E_{i}=\{\begin{array}{c} E_{i}^{t}+E_{i}^{c}\;\;\;\;\;\;\;\;\;\;\;\;\mathrm{if}\;i\;\mathrm{is}\;a\;\mathrm{source}\;\mathrm{node};\\ E_{i}^{t}+E_{i}^{c}+E_{i}^{r}\;\;\;\;\;\;\mathrm{if}\;i\;\mathrm{is}\;a\;\mathrm{relay}\;\mathrm{node};\\ E_{i}^{r}\;\;\;\;\;\;\;\;\;\;\;\;\;\mathrm{if}\;i\;\mathrm{is}\;a\;\mathrm{destination}\;\mathrm{node}; \end{array}$$

where $E_{i}^{t}$ = Pτ is the transmission energy consumed per bit, $E_{i}^{c}$ is the energy consumed per bit to activate the electronic circuitry and perform digital processing, and $E_{i}^{r}$ is the energy consumed per bit at the receiver. The total energy consumption *E*[*k*] per bit for a multicast session *k* is the sum of the energy consumed by each transceiver involved in the local forwarding operation in the multicast tree *T_k_*, which can be expressed as

(2) $$E\left[k\right]=\underset{\forall i\in T_{k}}{\sum }E_{i}$$

Let *T_G_* be a set of possible multicast trees for a session *k*, with each multicast tree *T_k_* having different relay nodes and multicast links. In this work, our focus is to select appropriate relay nodes and establish multicast links in such a way that the minimum energy multicast tree $T_{k}^{*}$ is constructed for each session *k*. This can be mathematically expressed as

(3) $$T_{k}^{*}=arg\;\;\begin{array}{c} \;min\\ \;\forall iT_{k}\in T_{G} \end{array}\;\underset{\forall i\in T_{k}}{\sum }E_{i}$$

While selecting the relay nodes and establishing the multicast links for a tree *T_k_*, we need to assign channels for each forwarding node which involves interference and scheduling constraints that needs to be obeyed.

Let us define a binary variable for the channel assignment

$$u_{ij}^{h}=\{\begin{array}{c} 1\\ 0 \end{array}\;\;\;\;\begin{array}{c} \mathrm{if}\;\mathrm{channel}\;h\;\mathrm{is}\;\mathrm{used}\;\mathrm{for}\;\mathrm{link}\;i\;\to \;j\;;\\ \mathrm{Otherwise} \end{array}$$

To avoid interference between hyperlinks, a hyperlink is established at a forwarding node *i* over channel *h* with *j* as one of the receiving nodes. Another hyperlink with forwarding node *p* and *q* as one of the receiving nodes cannot be established over the same channel if *d_pj_* < *R_T_* because the transmission of node *p* over the same channel may cause interference to the reception at node *j*. This can be mathematically expressed as

(4) $$u_{ij}^{h}+\underset{p\in Q_{j}}{\sum }u_{pq}^{h}\leq 1$$

where *Q_j_* is the set of nodes whose distance to node *j* is less than *R_T_* and which has channels in common with node *j*. Mathematically, *Q_j_* can be expressed as

*Q_j_* = {P: *d_pj_* < *R_T_*, and, *C_j_* ∩ *C_p_* ≠ Ø}

To summarize, we are a given set of sessions *K*. For each session *k* ∈ *K* we need to select the relay nodes and perform channel assignments to establish multicast links in such a way that a minimum energy multicast tree is formed for each session. Hence, our optimization problem has the objective function in (3) subject to the constraint in (4) and can be expressed as

(5) $$\begin{array}{c} \mathrm{Find}:T_{k}^{*}=arg\;\begin{array}{c} min\\ \forall iT_{k}\in T_{G} \end{array}\;\;\;\underset{\forall i\in T_{k}}{\sum }E_{i}\\ \mathrm{Subject}\mathrm{to}:u_{ij}^{h}+\underset{p\in Q_{j}}{\sum }u_{pq}^{h}\leq 1 \end{array}$$

According to [21], the problem of finding a minimum energy consumption-based multicast tree is an NP-complete problem. Thus, this study proposes a heuristic algorithm to solve this optimization problem.

## 4. Proposed Technique

The overall energy consumption *E*[*k*] for a multicast session *k* is the sum of energy consumed by the different local forwarding operations belonging to the multicast tree constructed for that session. Each forwarding operation can be represented by a hyperlink. Thus, *E*[*k*] for a multicast session *k* can be reduced by minimizing the number of local forwarding operations/hyperlinks. In multicast CR-WMNs, the hyperlinks for a multicast session can be minimized by covering more receiving nodes in a hyperlink, either by assigning a suitable multicast channel or selecting the forwarding nodes.

**Figure 1:**
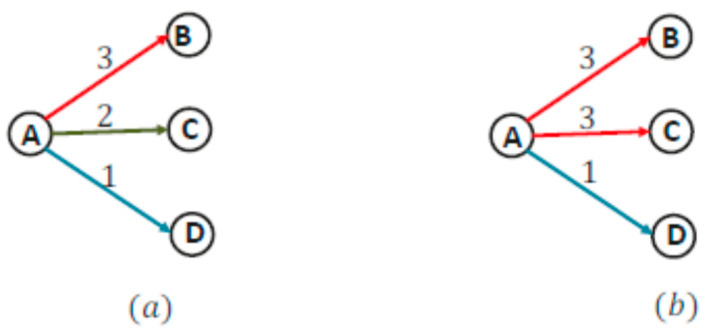
(**a**) Node A uses three channels {1, 2, 3}. (**b**) Node A uses two channels {1, 2}.

Figure 1a illustrates how channel assignment can help to reduce hyperlinks. Node A has four available channels, i.e., CA = {1, 2, 3, 4} and three neighboring nodes, i.e., B, C, and D. The available channels at nodes B, C, and D are {2, 3}, {3, 4}, and {1, 2}, respectively. The distance between node A and nodes B, C, and D means a single channel cannot be used to cover them because of channel heterogeneity. It can be observed that there does not exist a single channel that node A has in common with all three nodes. Thus, node A must use multiple channels to achieve this one hop multicast. Several different realizations are possible, among them, two are shown in Figure 1a,b. In Figure 1a, node A uses three channels (1, 2, 3), thus there are three hyperlinks, i.e., {{A, 3, {B}, {A, 2, {C,}, {A, 1, {D}}. However, Figure 1b uses two channels to cover all three nodes resulting in two hyperlinks, i.e., C_A_ = {1, 2, 3}; C_B_ = {2, 3}; C_C_ = {3, 4}; C_D_ = {1,2}.

According to the energy consumption model presented in (1), the energy consumed for a one hop multicast in Figure 1a is 3(*E^t^* + *E^c^*) + 4*E^r^*, whereas the energy consumed for the second case is 2(*E^t^* + *E^c^*) + 4*E^r^*. Hence, by assigning the appropriate channel, the number of hyperlinks can be reduced resulting in a reduction of overall energy consumption.

**Figure 2:**
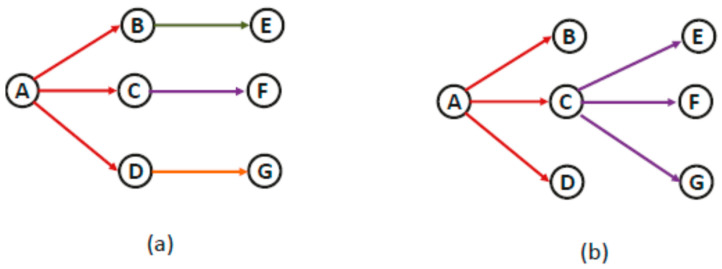
(**a**) A, B, C, and D are the forwarding nodes; (**b**) A and C are the forwarding nodes.

The example given in Figure 2 illustrates how the selection of forwarding nodes can help reduce hyperlinks in multicasts in CR-WMNs. Here, assume the same channels are available at each node and ignore spectrum heterogeneity. A multicast session with source node A and destination nodes E, F, and G is used. Assume that, based on distance, node B neighbors nodes E and F, node C neighbors nodes E, F, and G, node D neighbors nodes F and G, and vice versa. Any node neighbor to nodes E, F, and G can be a forwarding node for them based on which different realizations of hyperlinks are possible, out of which two are shown in Figure 2a,b. In Figure 2a, nodes B, C, and D are forwarding nodes for nodes E, F, and G, respectively, which results in three overall hyperlinks, i.e., {{B, 1, {E}, {{C, 2, {F}, {D, 3, {G}}, whereas there is only one hyperlink {{C, 1, {E, F, G} in Figure 2b for this one hop multicast. According to the energy consumption model presented in (1), the energy consumed for a one hop multicast in Figure 1a is 3(*E^t^* + *E^c^*) + 4*E^r^*, whereas the energy consumed in the second case is 2(*E^t^* + *E^c^*) + 4*E^r^*. Hence, by selecting the appropriate forwarding node, the number of hyperlinks can be reduced resulting in a reduction of overall energy consumption.

**Figure 3:**
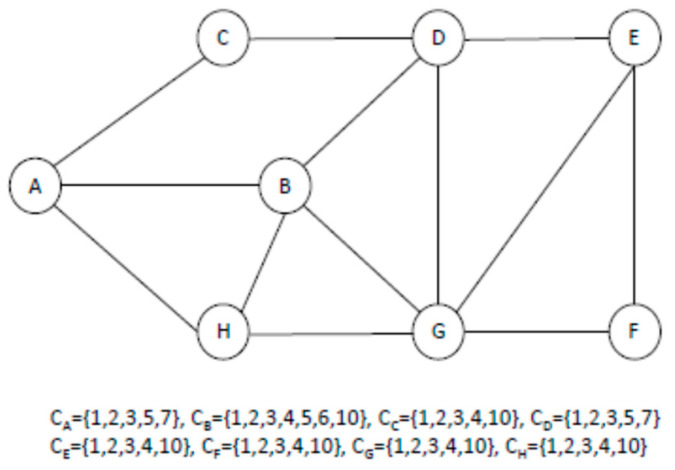
An Example of a CR-WMN.

**Figure 4:**
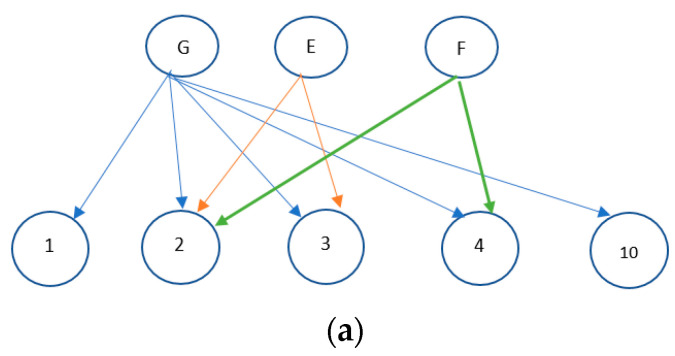

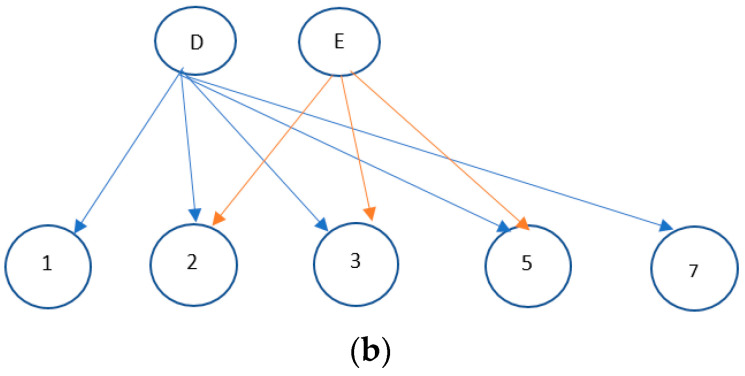
Bipartite graph for nodes G and D. (**a**) A bipartite graph for node G. (**b**) A bipartite graph for node D.

In this work, based on the joint consideration of the illustrated concepts, we proposed a heuristic algorithm to construct a minimum energy consumption-based multicast tree in CR-WMNs. For each session *k*, a node level is defined initially. Each node level is the least number of hops it has from the source node ranging from 0 to L, where level 0 is assigned to the source node and level L to the leaf nodes. For example, in Figure 3, node level is defined as ranging from L = 0 to L = 3. A is the source node and is assigned a level L = 0 and E and F are the leaf nodes and are assigned level L = 3. The tree construction then begins from level L. The forwarding nodes from level L1 are chosen for the potential receiving nodes at level L. For example, in Figure 3 at level L = 3, E and F are the potential receiving nodes and nodes at level L = 2., i.e., D and G, are the candidate forwarding nodes. A bipartite graph is created for each node at level L = 1 to choose the forwarding nodes for the potential receiving nodes of level L. For example, in Figure 3, a bipartite graph needs to be constructed for nodes D and G. A graph G(V, ξ) is called a bipartite graph if the set of vertices V can be partitioned into two disjoint sets, S_1_ and S_2_ with S_1_S_2_ = V, and if every edge in ξ connects a vertex from S_1_ to S_2_. G_i_ (S_1_, S_2_, ξ) is a bipartite graph, where S1 consists of node i and the potential receiving nodes of level L and S2 consist of the available channels at node i. For example, S1 for nodes G and D consist of nodes {G, E, F} and {D, E}, respectively. S1 for node D does not include F because the distance between nodes D and F is greater than RT. S_2_ for G and D consists of the respective channels available at each node. An edge (x, y) exists between vertices such that x ∈ S_1_ and y ∈ S_2_ if y ∈ C_x_, i.e., channel y is in the list of C_x_. Figure 4 shows the bipartite graph for nodes G and D. For G_i_ (S_1_, S_2_, ξ), find the number of edges finishing on each vertex of S_2_ which can be said to be the degree of that vertex. Subsequently, find the vertex with the maximum degree that represents the channel with which the forwarding node can cover the maximum possible potential number of receiving nodes. For example, in Figure 4a, the bipartite graph for node G, channel 2 has the maximum degree of 3. Subsequently, the same procedure is repeated for the remaining candidate forwarding nodes. Finally, the node with the maximum degree is chosen as the forwarding node and the corresponding channel is the selected channel. As illustrated in Figure 4, node G has channel 2 which has the maximum degree of 3, and node D has three channels (2, 3 and 5) which have a degree of 2. Since node G has a channel with the maximum degree, it is selected as the forwarding node and it is assigned to channel 2, which ultimately results in the formation of a hyperlink {{G, 2, {E, F}}. Alternatively, if node D is selected as the forwarding node, it cannot cover both nodes E and F. Thus, this mechanism results in the formation of a hyperlink that can cover the maximum number of possible potential receiving nodes. The algorithmic form of this mechanism is given in Algorithm 1. Once a channel is assigned to a hyperlink, all nodes whose distance to any of the nodes involved in this hyperlink (either forwarding or receiving node) is less than R_T_ remove this channel from the list of available channels to ensure the constraint mentioned in (4). Next, if all the potential receiving nodes of level L are covered, a search for forwarding nodes in level L1 is performed by following the same procedure. As shown in Figure 3, both potential receiving nodes, E and F, are covered by the selected forwarding node G. Otherwise, remove the covered nodes from the list of potential receiving nodes and again execute Algorithm 1 repeatedly until all potential receiving nodes are covered.
**Algorithm 1**INPUT: G(S_1_ ∪ S_2_, ξ), A bipartite graph for node i
    N_L_, Number of nodes in level L
    OUTPUT: H(h, Y, Q)
    Where h is the forwarding node;
    Y is the selected channel;
    and Q is set of covered nodes.
    Initialization
    I = 1;
    X_i_ = S_i_;
    i ≤ N_L_ Find y∗ = arg max deg(y)
    y ∈ C_i_
    X_i_ = X_i_ ∩ U_i_,
    U_i_ = {j ∈ S|y∗ ∈ C_j_}
    |X_i_| > |Q_i_| Q_i_ ← X_i_
    Y_i_ ← y∗
    h ← i
    Return H(h,Y,Q)

## 5. Simulation Results

In this section, simulation results of the proposed technique are presented. Performance is measured in terms of average energy consumption per bit for a multicast session and the average of engaged channels. Average energy consumption is only measured for the data packet, whereas the energy consumed for communicating the spectrum sensing results over the control channel is not considered. Engaged channels indicate the number of channels used among the total available channels. All simulations were carried out in MATLAB and the Monte Carlo simulation technique is carried out to report the average results. In order to evaluate the performance of the proposed technique, a comparison is made with the flooding approach used for multicast transmission. Six parameters are primarily used in the simulation, including the number of levels/hops denoted by L, the number of destination nodes denoted by D, the number of channels denoted by M, the probability of channels being available denoted by σ, the total energy denoted by *ET*, and the number of channels being used denoted by *K*. Performance is evaluated in terms of the average number of channels used K and the average energy consumed ET for a multicast session. M and σ are set to 100 and 0.7, respectively. The parameters D and L vary from 2 to 10 and 4 to 6, respectively.

**Figure 5:**
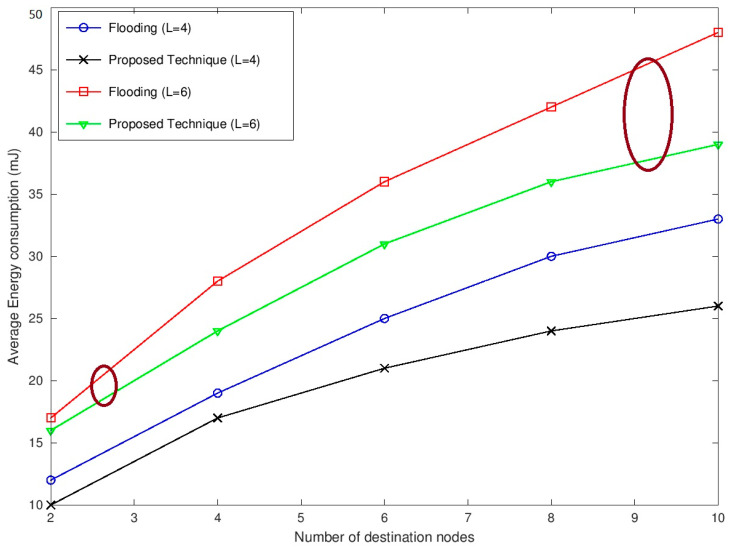
Average energy consumption versus the number of destination of nodes.

Figure 5 shows the average energy consumed during multicast sessions for L = 4 and L = 6 by varying the number of destination nodes from 2 to 10. It can be observed that with an increase in the number of nodes, the energy consumed is also increasing. The curves for L = 4 and L = 6 also show a clear variation in the amount of energy consumed. As far as the comparison between the flooding technique and the proposed technique is concerned, the proposed technique consumes less energy. Less energy consumption occurs due to the minimum number of transmissions as well as the selection of channels which requires less energy. In addition, the proposed technique does not have to relay the data to every node in the network. Consequently, the energy consumed by the proposed technique is significantly less than the energy consumed by the flooding technique for setting up a multicast session.

**Figure 6:**
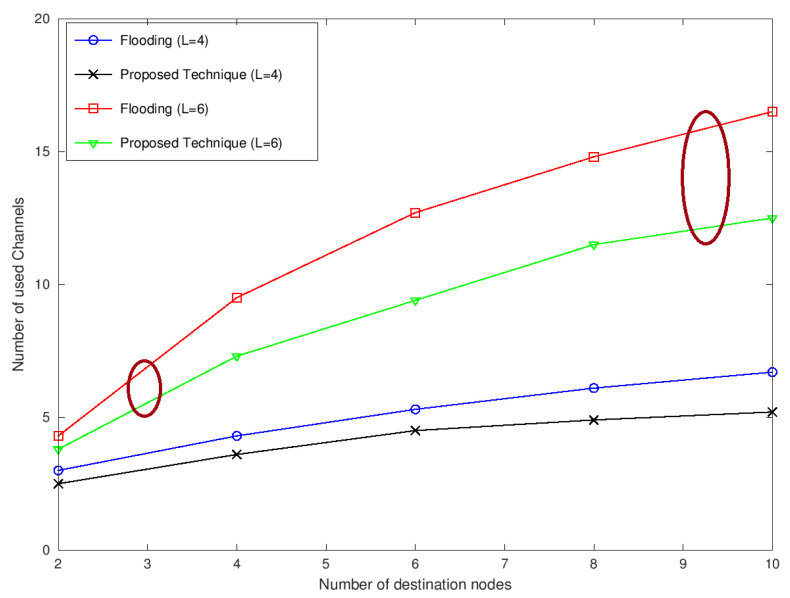
Average number of used channels versus the number of destination nodes.

Figure 6 shows the average number of channels used for L = 4 and L = 6 by varying the number of destination nodes from 2 to 10. It is apparent that with an increase in the number of nodes, the number of channels used is also increasing. The curves for L = 4 and L = 6 also show the difference in the average number of channels used for multicast sessions. While comparing the flooding technique and the proposed technique, it can be observed that the proposed technique utilizes significantly fewer channels because the flooding approach has to relay the data to every other node in the network. In contrast, the proposed technique only sends the data to the selected nodes. The selection of relaying nodes is based on the minimum number of data transmissions to the maximum possible nodes in the next level. This is possible only when the selected channel is available to more and more nodes. This channel selection technique results in the utilization of fewer channels compared to the flooding approach.

**Figure 7:**
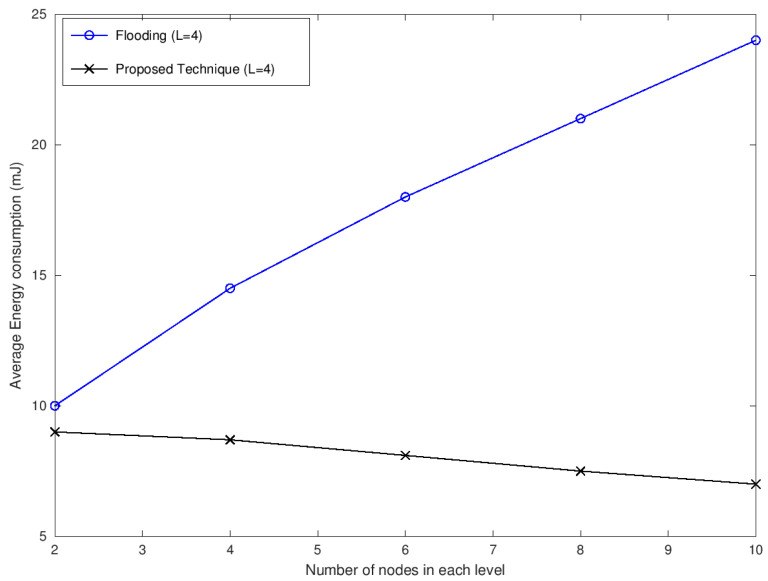
Average energy consumption versus the number of destination nodes.

Figure 7 shows the average energy consumption of the multicast session against the varying number of relaying nodes at each intermediate level. The first level consists of one node called the source of the multicast session. The last level consists of four nodes that are supposed to receive the data and are called the destination. There are four levels in the current study, which means that there are two intermediate levels, and the nodes in each of these levels varies from 2 to 10. There is a significant variation in energy consumption between the proposed technique and the flooding approach. With an increase in the number of nodes at intermediate levels, energy consumption increases for the flooding approach while it decreases in the proposed approach. The number of transmissions for the flooding approach increases with an increase in the number of nodes at the intermediate level. Contrastingly, with an increase in the number of nodes at the intermediate level, the number of transmissions for the proposed approach decreases. This is because a larger pool of candidates is available to be selected as a relay node. Consequently, a relay node that covers a large number of data receiving nodes in the next level has been selected for transmission. This results in a reduced number of transmissions and therefore reduces energy consumption.

## 6. Conclusions

An energy efficient channel assignment and relay node selection problem for multicasting in CR-WMNs for multimedia transmission has been formulated. To achieve this objective, the number of transmissions should be reduced. For this purpose, two methods were chosen to reduce (1) the number of forwarding/relaying nodes and (2) the number of channels used for transmission. As a result, the energy consumption of a particular multimedia session is reduced. In order to select the forwarding nodes and channels for multimedia transmission, we proposed a heuristic algorithm in which, first and foremost, a bipartite graph for each forwarding node is generated. This bipartite graph consists of two sets. One set includes all the nodes to which the data must be forwarded and the node that will forward. The second set consists of all the channels available with these nodes. A graph is extracted from this in such a way that the product of the number of channels and nodes is maximized. The same method is repeated for all nodes and the node with the largest value is chosen as the forwarding node. The hyperlink is generated based on the method mentioned above. This method is repeated for all remaining nodes that must forward the data and are uncovered by this hyperlink as well as for all other levels. This process starts at the last level and goes back to the first level. As a result, a tree is formed that allows the operation of a multicast session with the minimum number of relaying nodes and channels that eventually reduces the number of transmissions and the energy consumed. The result has been compared to the flooding approach and we observed a significant drop in energy consumption and the number of channels used while varying the number of destination nodes. In the future, this work can be extended to multiple sessions running simultaneously. Furthermore, this work is limited to data packet energy consumption, which can be broadened by incorporating control information energy consumption, including spectrum sensing results communicated among neighboring nodes prior to selecting relay nodes and forwarding channels.

## Figures and Tables

**Table 1:** Summary of Related Work.

Ref	Year	Objective	CR	WMN	Multicast	Approach	Remarks
[6]	2015	Automata based multipath multicasting in CRN	✓		✓	A multi-path on-demand routing protocol based on the Artificial Intelligence Reinforcement Learning Method	Increased throughput and reduced end-to-end delays
[7]	2010	Assisted-multicast scheduling in wireless cognitive mesh networks	✓	✓	✓	A multicast scheduling technique where client nodes assist the base station in delivering the multicast data over orthogonal channels	Reduced the total multicast time
[8]	2017	Spectrum-aware network coded multicast in mobile cognitive radio adhoc networks	✓		✓	A multicast scheduling technique where client nodes assist the base station in delivering the multicast data over orthogonal channels	Reduced the total multicast time
[9]	2018	Packet multicast in cognitive radio adhoc networks	✓		✓	Random network coding based multicasting strategy	Reduced the total number of transmissions
[10]	2010	Multicast scheduling with cooperation and network coding in CRN	✓		✓	Optimization method for multicast scheduling using network coding	Protected the PU and fairness
[11]	2013	Channel allocation and multicast routing in CRN	✓		✓	Optimization problem which jointly defines multicast routes and channel assignments	Increased multicast throughput in multi-hop CRNs
[12]	2017,	Design of cooperative non-orthogonal multicast cognitive multiple access	✓		✓	Implementation of multicast non-orthogonal multiple access (NOMA) in CRNs	Significant performance gains were achieved
[13]	2018,						
[14]	2017						
[15]	2012	QoS multicast routing in cognitive radio adhoc networks	✓		✓	A multicast tree construction method based on a minimal spanning tree	Reduced bandwidth usage
[16]	2016	Minimum spanning tree-based multicast routing protocol for dynamic spectrum access networks	✓		✓	A minimum tree-based multicast routing protocol is proposed	Increased total throughput and increased the delivery rate of packets
[17]	2013	Joint power and rate allocation for spectrum sharing cognitive radio multicast networks under service outage constraint	✓		✓	Joint power and rate allocation	Maximized the average multicast rate in CRNs
[18]	2018	An efficient channel assignment algorithm for multicast wireless mesh networks		✓	✓	Local multicast concept and a channel assignment algorithm	Considered both partially overlapping as well as orthogonal channels
[19]	2018	Interference optimization for multicast and broadcast traffic in multi-radio multi-channel WMNs equipped with directional antennas		✓	✓	Considered multi-channel multicast problem for a multi-radio network of wireless mesh nodes with directional antennas	Reduced interference

## References

[1] Arjoune Y., Kaabouch N. (2019). A Comprehensive Survey on Spectrum Sensing in Cognitive Radio Networks: Recent Advances, New Challenges, and Future Research Directions. Sensors.

[2] Amjad M., Rehmani M.H., Mao S. (2018). Wireless Multimedia Cognitive Radio Networks: A Comprehensive Survey. IEEE Commun. Surv. Tutor..

[3] Raghu R., Upadhyaya P., Panju M., Agarwal V., Sharma V. (2019). Deep Reinforcement Learning Based Power Control for Wireless Multicast Systems. Proceedings of the 2019 57th Annual Allerton Conference on Communication, Control, and Computing (Allerton).

[4] Liu Q., Ren H.-P., Tang R.-J., Yao J.-L. (2020). Optimizing Co-Existing Multicast Routing Trees in IP Network via Discrete Artificial Fish School Algorithm. Knowl. Based Syst..

[5] Basaras P., Iosifidis G., Kucera S., Claussen H. (2020). Multicast Optimization for Video Delivery in Multi-Rat Networks. IEEE Trans. Commun..

[6] Ali A., Qadir J., Baig A. (2015). Learning Automata Based Multipath Multicasting in Cognitive Radio Networks. J. Commun. Netw..

[7] Almasaeid H.M., Kamal A.E. (2010). Assisted-Multicast Scheduling in Wireless Cognitive Mesh Networks. Proceedings of the 2010 IEEE International Conference on Communications.

[8] Qu Y., Dong C., Guo S., Tang S., Wang H., Tian C. (2016). Spectrum-Aware Network Coded Multicast in Mobile Cognitive Radio Ad Hoc Networks. IEEE Trans. Veh. Technol..

[9] Chen B., Gao Z., Yang M., Ning Q., Yu C., Pan W., Nian M., Xie D. (2018). Packet Multicast in Cognitive Radio Ad Hoc Networks: A Method Based on Random Network Coding. IEEE Access.

[10] Jin J., Xu H., Li B. (2010). Multicast Scheduling with Cooperation and Network Coding in Cognitive Radio Networks. Proceedings of the 2010 Proceedings IEEE INFOCOM.

[11] Shu Z., Qian Y., Yang Y., Sharif H. (2013). Channel Allocation and Multicast Routing in Cognitive Radio Networks. Proceedings of the 2013 IEEE Wireless Communications and Networking Conference (WCNC).

[12] Lv L., Chen J., Ni Q., Ding Z. (2017). Design of Cooperative Non-Orthogonal Multicast Cognitive Multiple Access for 5G Systems: User Scheduling and Performance Analysis. IEEE Trans. Commun..

[13] Bhattacharjee S., Acharya T., Bhattacharya U. (2018). NOMA Inspired Multicasting in Cognitive Radio Networks. IET Commun..

[14] Chen Y., Wang L., Jiao B. (2017). Cooperative Multicast Non-Orthogonal Multiple Access in Cognitive Radio. Proceedings of the 2017 IEEE International Conference on Communications (ICC).

[15] Xie L., Jia X., Zhou K. (2012). QoS Multicast Routing in Cognitive Radio Ad Hoc Networks. Int. J. Commun. Syst..

[16] Al-rubaye M., Salameh H.B., Jararweh Y. (2016). Minimum Spanning Tree-Based Multicast Routing Protocol for Dynamic Spectrum Access Networks: A Multi-Layer Probabilistic Approach. Proceedings of the 2016 7th International Conference on Computer Science and Information Technology (CSIT).

[17] Xu D., Feng Z., Wang Y., Zhang P. (2013). Joint Power and Rate Allocation for Spectrum Sharing Cognitive Radio Multicast Networks under Service Outage Constraint. AEU-Int. J. Electron. Commun..

[18] Shi W., Wang S., Wang Z., Wang E. (2018). An Efficient Channel Assignment Algorithm for Multicast Wireless Mesh Networks. AEU-Int. J. Electron. Commun..

[19] Avokh A., Mirjalily G. (2018). Interference Optimization for Multicast and Broadcast Traffics in Multi-Radio Multi-Channel WMNs Equipped with Directional Antennas. AEU-Int. J. Electron. Commun..

[20] Dong C., Qu Y., Dai H., Guo S., Wu Q. (2018). Multicast in Multi-Channel Cognitive Radio Ad Hoc Networks: Challenges and Research Aspects. Comput. Commun..

[21] Čagalj M., Hubaux J.-P., Enz C. Minimum-Energy Broadcast in All-Wireless Networks: NP-Completeness and Distribution Issues. Proceedings of the 8th Annual International Conference on Mobile Computing and Networking.

